# Metabolic engineering of biocatalysts for carboxylic acids production

**DOI:** 10.5936/csbj.201210011

**Published:** 2012-11-12

**Authors:** Ping Liu, Laura R. Jarboe

**Affiliations:** a Department of Chemical and Biological Engineering, Iowa State University, Ames, Iowa, USA

**Keywords:** metabolic engineering, carboxylic acid production, tolerance

## Abstract

Fermentation of renewable feedstocks by microbes to produce sustainable fuels and chemicals has the potential to replace petrochemical-based production. For example, carboxylic acids produced by microbial fermentation can be used to generate primary building blocks of industrial chemicals by either enzymatic or chemical catalysis. In order to achieve the titer, yield and productivity values required for economically viable processes, the carboxylic acid-producing microbes need to be robust and well-performing. Traditional strain development methods based on mutagenesis have proven useful in the selection of desirable microbial behavior, such as robustness and carboxylic acid production. On the other hand, rationally-based metabolic engineering, like genetic manipulation for pathway design, has becoming increasingly important to this field and has been used for the production of several organic acids, such as succinic acid, malic acid and lactic acid. This review investigates recent works on *Saccharomyces cerevisiae* and *Escherichia coli*, as well as the strategies to improve tolerance towards these chemicals.

## Introduction

Carboxylic acids can be used as platform chemicals to generate primary building blocks of industrial chemicals by both enzymatic and chemical catalysis. For example, free fatty acids can be extracted from the fermentation medium and catalytically converted into esters or alkanes (54, 71). As the demand for sustainable energy increases, production of useful chemicals from renewable feedstocks using biocatalyst fermentation is more attractive as a replacement for petroleum-based fuels and chemicals. Currently, several carboxylic acids have been fermentatively produced (Table 1). However biocatalysts with high product yield, titer and productivity are desirable in order for fermentative processes to be economically competitive with petroleum-based processes (2, 34).

**Table 1:** Production of the carboxylic acids malate, lactate and succinate by *E. coli* and *S. cerevisiae* from glucose.

Carboxylic acid	Organism	Condition	Titer (g/L)	Yield (g/g)	Productivity g/L/h	Refs
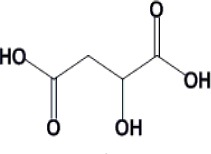 Malate	*S. cerevisiae*	Aerobic flask	59	0.31	0.19	(82)

	*E. coli*	Two-stage process	34	1.05	0.47	(85)

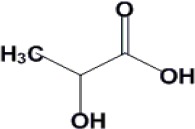 Lactate	*S. cerevisiae*	Anaerobic, batch	70	n/a	0.93	(72)

	*E. coli*	Anaerobic, batch	118	0.98	2.88	(24)

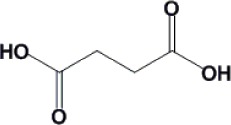 Succinate	*S. cerevisiae*	Shake flask	3.62	0.1	n/a	(62)

	*E. coli*	Anaerobic, batch	83	0.92	0.88	(32)

n/a – not available

Selection-based strain improvement, often enabled by random mutagenesis, has been very successful for the production of carboxylic acids (5, 7). However, our ability to produce carboxylic acids and other fermentation products is often limited by complex cellular metabolism and regulations (20). Currently, as information is acquired from new technologies such as high- throughput genomic sequencing and DNA recombination technology, we have the ability to overcome these limitations and improve microbial performance by fine-tuning enzymatic, transport and regulatory functions (8). Metabolic engineering, defined as “*the directed improvement of production, formation, or cellular properties through the modification of specific biochemical reactions or the introduction of new ones with the use of recombinant DNA technology”* plays a key role in improving strain performance (22, 37).

**Figure 1:**
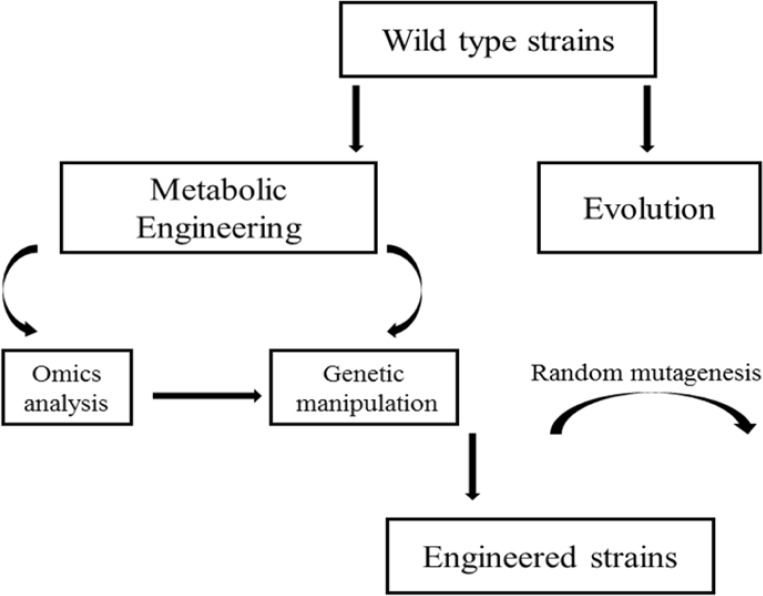
Strain development methods in carboxylic acid production

Here, we describe the use of metabolic engineering, motivated and guided in part by omics analysis, to enable desirable microbial performance for fermentative production of carboxylic acids (Figure 1). We mainly focus on recent progress with *Escherichia coli* and *Saccharomyces cerevisiae* for production of lactic acid, malic acid and succinic acid. *S. cerevisiae* is appealing for carboxylic acids production because it can tolerate low pH. This reduces the need for maintenance of neutral pH via alkali addition and the low-pH fermentation broth is less vulnerable to contamination. Moreover, product tolerance can be another key factor in regards to the performance of developed strains, so strategies to improve tolerance to carboxylic acids are also discussed.

## 1. Metabolic Engineering by genetic manipulations

### 1.1 Improvement of product formation by overexpression of key pathway enzymes

**Figure 2:**
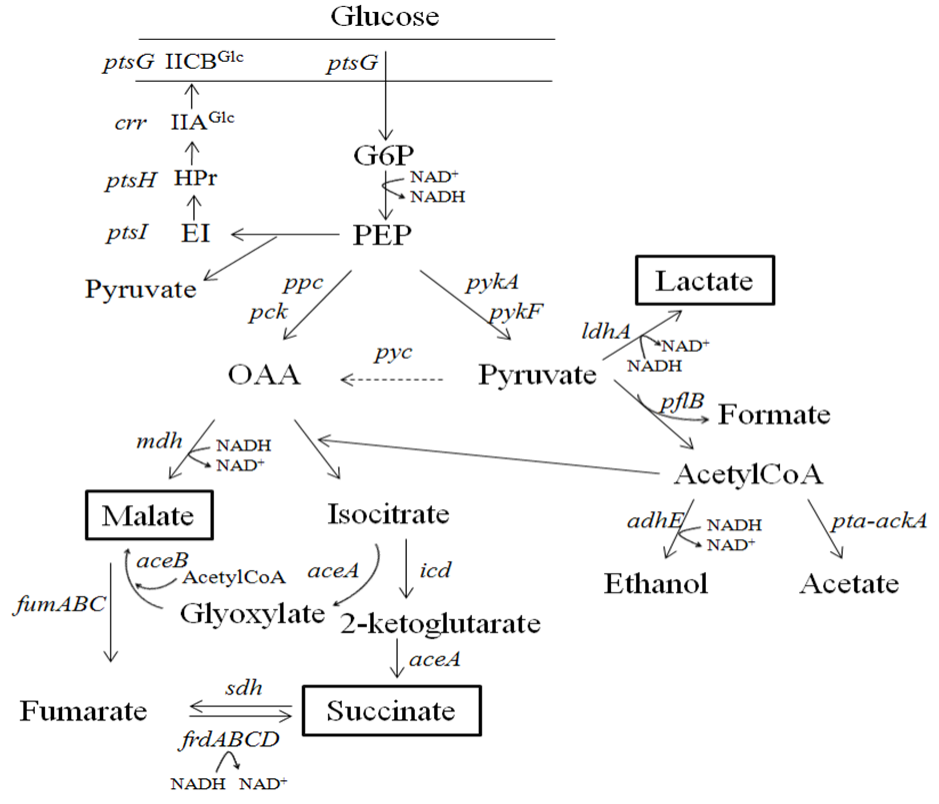
Metabolic pathways for production of lactate, malate and succinate in *E. coli*. For simplicity, cofactor usage is not shown. Heterologous genes expression is indicated by a dashed line. Genes and enzymes: *aceA*, isocitrate lyase; *aceB*, malate synthase; *ackA*, acetate kinase; *adhE*, aldehyde dehydrogenase; *crr*, glucose-specific phosphotansferase enzyme IIA component; *fumABC*, fumarase isoenzymes; *frdABCD*, fumarate reductase; *icd*, isocitrate dehydrogenase; *ldhA*, lactate dehydrogenase; *mdh*, malate dehydrogenase; *ppc*, phosphoenolpyruvate carbolxylase (PEPC); *pck*, phosphoenolpyruvate carboxykinase (PEPCK); *pyc*, pyruvate carboxylase (PYC); *pykA and pykF*, pyruvate kinases; *pflB*, pyruvate-formate lyase; *pta*, phosphate acetyltransferase; *ptsG*, PTS system glucose-specific EIICB component; *ptsH*, phosphocarrier protein HPr; *ptsI*, phosphoenolpyruvate-ptotein phosohotransferase; *sdh*, succinate dehydrogenase.

Increasing the expression of key enzymes in the desired metabolic pathway, as well as deletion of competing pathways, is often necessary to improve target production. There are many examples of this type of strategy enabling production of carboxylic acids. In this section, we review overexpression of both native and heterologous enzymes contributing to improved succinate production by *E. coli* and malate production by *S. cerevisiae*; Figure 2 shows a simplified overview of central carbon metabolism in *E. coli*.

Under anaerobic conditions, the formation of succinic acid by *E. coli* is primarily from the carboxylation of phosphoenolpyruvate (PEP) into oxaloacetate (OAA). This pathway is encoded by two enzymes: PEP carboxylase (PEPC, encoded by *ppc*) and PEP carboxykinase (PEPCK, encoded by *pck*). Overexpression of *ppc* has been reported to significantly increase succinic acid production from glucose (50). However, no effect was found by overexpression of the native PEPCK in *E. coli* (50). Furthermore, overexpression of PEPCK from *Actinobacillus succinogenes*, the main CO_2_-fixing enzyme in the *A. succinogenes* succinate production pathway, in *E. coli ppc-*deficient mutant strains increased the production of succinic acid by as much as 6.5-fold (38).

In *E. coli*, PEP may also be converted into pyruvate either by the phosphotransferase system (PTS) or by pyruvate kinase. In other organisms, pyruvate can be converted into OAA by pyruvate carboxylase (PYC) (6, 59), which is not present in *E. coli*. Therefore, another way to produce more OAA is by the heterologous expression of pyruvate carboxylase. The *pyc* gene from *Rhizobium etli* was expressed in *E. coli*, leading to an increase in succinate production from 1.18gL^-1^ to 1.77g L^-1^ (23). Co-overexpression of genes encoding PEPC from *Sorghum vulgare* and PYC from *Lactococcus lactis* in *E. coli* increased the succinic acid yield relative to individual overexpression of only PEPC or PYC (47).

In succinate production by *E. coli*, NADH availability was reported to be a limiting factor. The fermentative pathway converting OAA to succinate requires 2 moles of NADH per succinate produced. However, one mole of glucose can only provide 2 moles of NADH through the glycolytic pathway. So the maximum theoretical yield of succinate is one mole per glucose consumed (65). The improved succinate yield can be accomplished by increasing availability of NADH. Berríos-Rivera et al heterologously expressed NADH-forming formate dehydrogenase from *Candida boidinii* in *E. coli* to generate 4 moles NADH per glucose consumed. Futhermore, this strategy was improved to produce more than 4 moles of NADH per glucose by combination with a more reduced carbon source (9). Additionally, a novel pathway with a reduced stoichiometric NADH/succinate molar ratio has been reported to increase succinate yield and productivity in *E. coli*. Three genes (*ldhA*, *adhE*, *ack-pta*) involved in central anaerobic pathway and one gene (*iclR*) involved in regulation of the glyoxylate pathway under aerobic conditions were deleted to eliminate competing NADH pathways and redirect carbon flux through the fermentative pathway and the glyoxylate pathway. Additionally, pyruvate carboxylase from *Lactococcus lactis* was expressed in the above mutant at the same time to increase the flux from pyruvate to OAA. The resulting strain can efficiently produce 1.61 moles of succinate per mole glucose, with only 1.25 mole of NADH needed (65).

Wild-type *S. cerevisiae* can naturally produce low levels of L-malate as this compound is part of the central metabolic pathways, such as the TCA cycle. Although four pathways have been identified in *S. cerevisiae* for malate formation, the most promising route for malate production from glucose is from pyruvate followed by reduction of OAA to malate, resulting in a maximum theoretical yield of 2 mol of malate per mol of glucose. This pathway involves the cytosolic enzymes pyruvate carboxylase and malate dehydrogenase (82). Overexpression of the cytosolic isoenzyme of malate dehydrogenase (Mdh2p) increased malate production to 12 g L^-1^ (61), but Mdh2p is subject to repression by glucose, both at the enzyme and transcript level (51). Furthermore, the strategy for cytosolic malate dehydrogenase overexpression was based on retargeting the peroxisomal isoenzyme encoded by *MDH3* to the cytosol by deletion of the C-terminal peroxisomal targeting sequence. This strategy increased the malate concentration more than 3-fold in shake flask experiments. However, overexpressing pyruvate carboxylase (*PYC2*) did not significantly improve malate production (82).

Malate transport is also an important strategy to improve malate production. *S. cerevisiae* does not have a membrane transporter for malate and the diffusion across the plasma membrane is slow (74). Thus, there has been interest in the use of heterologous transporters.

Expression of the malate transporter SpMAE1 from yeast *Schizosaccharomyces pombe* was first reported to mediate the import of malate in *S. cerevisiae* (75); later studies showed that expression of SpMAE1 also enabled increased malate production (14, 82). Moreover, simultaneous overexpression of the native pyruvate carboxylase, cytosolic malate dehydrogenase and SpMAE1 in *S. cerevisiae* generated a high malate-producing strain with titers of 59 g L^-1^ and a malate yield of 0.42 mol per mol glucose (82).

### 1.2 Improvement of product formation by inactivation of competing pathways

Deletion of metabolic pathways that compete with production of the target compound can be a useful method for redirecting metabolic flux into the desired pathway.

Anaerobic production of succinate by *E. coli* is normally associated with co-production of acetate, formate, lactate and ethanol. Preventing the formation of these byproducts would improve succinate production by both increasing product purity and hopefully increasing product yield and concentration, though this is challenging given the constraints of maintaining redox balance and the need for a net generation of ATP. Deletion of lactate dehydrogenase (*ldh*) eliminates the pathway that converts pyruvate to lactate (48). Formation of the other three byproducts (ethanol, formate and acetate) is dependent on pyruvate formate lyase, which converts pyruvate into acetyl-CoA and formate. Although simultaneous inactivation of the pyruvate-formate lyase (*pflB*) and lactate dehydrogenase (*ldhA*) resulted in the intended decrease in production of lactate, acetate and ethanol, unfortunately this double mutant strain was unable to ferment glucose. However, a spontaneous mutation in this *ΔpflB ΔldhA* strain restored its ability to ferment glucose and produce succinic acid, acetic acid and ethanol in proportions of 2:1:1, which was an improvement relative to the wild-type ratio of 1:2:2 (21). Furthermore, the causative mutation restoring glucose fermentation was mapped to the *ptsG* gene encoding a membrane-bound, glucose-specific permease in the phosphotransferase system (PTS). Specifically, inactivation of the *ptsG* gene in the double mutant strain restored the ability to ferment glucose and increased succinic acid production (18). Redox balance is also an important factor in metabolic engineering and strain development. The double mutant (*ΔpflB ΔldhA*) resulted in a NADH/NAD^+^ 2:1 imbalance, which can limit growth. Singh et al identified a series of genes related to NADH oxidation: *grxB*, *hyfF*, *yhcA*, *argA*, *pfkB*, *marA*, *moaE*, *ygfT*, and *nuoC*. Overexpression of these genes improved the growth of a double mutant with reduced NADH/NAD^+^ ratio and improved succinate production up to 20% in minimal media plus 10g/L glucose (67).

Reducing the metabolic flux to pyruvate is also critical for succinic acid production. Triple deletion mutants for three pyruvate-forming enzymes (*ptsG*, *pykF* and *pykA*) produced 2.05 g L^-1^ succinic acid, a more than sevenfold increase over the wild type (0.29 g L^-1^) (41).

Under aerobic conditions, the most effective way to produce succinic acid is through the glyoxylate cycle, in which isocitrate is converted into succinate and glyoxylate by isocitrate lyase (*aceA*). Disruption of succinate dehydrogenase (*sdh*), isocitrate dehydrogenase (*icd*), glyoxylate operon *aceBAK* repressor (*iclR*) and acetate pathways (*poxB, ackA-pta*) redirected the carbon flux through the glyoxylate bypass, resulting in production of 5.08 g L^-1^ (43mM) succinate in an aerobic batch fermentation (46). The same strategy was applied in yeast: genes encoding succinate (*SDH1, SDH2*) dehydrogenase and isocitrate dehydrogenase (*IDH1*, *IDP1*) were deleted from *S. cerevisiae*, increasing succinate production from 0.76 g L^-1^ to 3.62 g L^-1^ (62).

Ethanol is often produced as an undesirable byproduct during carboxylic acid production by yeast. There are two enzymes associated with ethanol production: pyruvate decarboxylase (PDC) and alcohol dehydrogenase (ADH). The first attempt to eliminate ethanol formation was conducted in a lactate-producing strain. The *ADH1* gene encoding ADH, which converts acetaldehyde into ethanol, was deleted. However, the decreased ethanol titer in the *adh1*-deletion strain did not result in increased accumulation of lactate (68). While deletion of all three *PDC* genes (*PDC*1, 5 and 6) encoding PDC isozymes completely eliminated ethanol formation and increased the accumulation of pyruvate, the mutant strains showed growth defects when grown on glucose as the sole carbon source. This weakness was addressed by directed evolution of a *PDC* knock-out strain using glucose as sole carbon source (73).

## 2. Omics analysis

Although genetic manipulation is powerful, its application is limited to previously-characterized enzymes and regulators. Omics analysis can provide the global information from disturbed metabolism and find the potential target genes for problem solving.

### 2.1 Transcriptome analysis

Transcriptome analysis, either by DNA microarray or sequencing-based quantification, has proven to be a powerful tool in the identification of novel target genes for improving strain performance (26).

One of the successful examples was performed for lactate production. In order to further improve lactate production by *S. cerevisiae*, the whole gene expression data was compared between a L-lactate-producing strain that expressed the human L-lactate dehydrogenase and the same strain harboring an empty plasmid. One of the most notable differences between the engineered and control strains was a 28-fold increase in abundance of the L-lactate cytochrome-c oxidoreductase encoded by *CYB2* gene in the engineered strain. In *S. cerevisiae*, the function of *CYB2* is to oxidize lactate to pyruvate. Its high expression suggested that some of the lactate was being assimilated back to pyruvate in the engineered strain and prevention of this assimilation could increase lactate production. Subsequent deletion of the *CYB2* gene confirmed this hypothesis by increasing L-lactate production 1.5-fold (57).

The usefulness of transcriptome analysis in the identification of targets for metabolic engineering was further demonstrated by a microarray-based selection and screening of deletion strains. Lactate dehydrogenase (LDH) is the enzyme responsible for lactate production. Gene expression profiles were compared between the L-lactate producing strain (carrying LDH from human) and its control strain (carrying the plasmid without LDH). This analysis identified 388 genes with significantly altered abundance in the L-lactate producing strain. In order to verify the effectiveness of microarray-based selection, individual deletions for 289 of these genes, as well as deletions for 56 randomly selected genes, were implemented into the strain with the plasmid carrying the human LDH gene. The lactate productivity was compared between these two groups of deletion strains and a control strain without the human LDH gene. Significantly altered L-lactate production was observed in 59 of the deletion strains selected based on the transcriptome data and in none of the 56 randomly-selected strains (27).

Regulators controlling the pathway for target production can also be identified from transcriptome analysis. The Hap2/3/4/5 complex activates transcription of almost all genes involved in TCA cycle, oxidative phosphorylation and respiration (55). Hap4p is mainly responsible for the activation of transcription produced by this complex (56). Yano et. al. found that *HAP4* is related to the production of malate and succinate in *S. cerevisiae* (81). A yeast strain (2OG-R39) with high malate and succinate production was isolated by mutagenesis of its parental strain (K-701). By comparing the transcriptome profiles of these two strains, the genes involved in TCA cycle, oxidative phosphorylation and respiration were found to be upregulated in strain 2OG-R39. Furthermore, a Northern blot analysis confirmed that *HAP4* had increased transcript abundance in strain 2OG-R39 than its parent strain. Subsequent productivity tests showed that overexpression of *HAP4* resulted in increased production of malate and succinate.

### 2.2 Proteomics

Proteomics examines the levels of proteins and their changes under particular genetic and environmental conditions, providing the information of complicated biological processes and posttranslational modifications (25).

The use of pentose sugars, such as xylose, as fermentation feedstocks remains challenging because many biocatalysts cannot use it as a carbon source. Although *E. coli* can naturally metabolize xylose to produce D-lactate, limitations of efficient xylose utilization still exist. In order to increase the lactate production from xylose, genes involved in competing pathways (*pflB*, *adhE* and *frdA*) and an ATP-dependent xylose transporter (encoded by *xylFGH*) were deleted from wild type *E. coli* MG1655 to generate strain JU01. Furthermore, an adaptive evolution with increasing xylose as the sole carbon source was performed with JU01 to generate the robust strain JU15. JU15 had a 2.7-fold increase in xylose consumption rate and 19-fold increase in lactate yield relative to wild type *E. coli*. To identify the mechanism of the increased xylose utilization, quantitative proteomics were used to compare the parental strains and the evolved strain. The results showed increased abundance of most of the enzymes involved in glycolytic pathways and xylose consumption, suggesting a change in a xylose transporter for a higher catabolism of xylose. Further investigation of the evolved strain JU15 using comparative genome sequencing and phenotypic validations identified *gatC* as a xylose transporter. In strain JU15, a point mutation within *gatC*, which resulted in a change from serine to leucine at position 184, is responsible for the high xylose consumption phenotype (35). Note that GatC has been reported as the IIC component of galactitol PTS system.

*Mannheimia succiniciproducens* has been reported to produce relatively large amounts of succinic acid under CO2-rich conditions (69). In order to elevate the production of succinate, the genes encoding lactate dehydrogenase, pyruvate-formate lyase, phosphotransacetylase, and acetate kinase were deleted from strain *M. succiniciproducens* MBEL55E; the resulting strain was named LPK7. Proteomic analysis, performed with both two-dimensional gel electrophoresis and mass spectrometry, was used to compare LPK7 to its parent strain MBEL55E in both exponential and stationary phase. This analysis revealed altered expression of enzymes involved in ATP formation and consumption, pyruvate metabolism, glycolysis and amino acid biosynthesis. Additionally, the changes in amino acid biosynthesis are important to illustrate why LPK7 can produce more succinic acid than its parent strain. The starting C4-compound for succinic acid production is oxaloacetate (OAA). Overexpression of genes catalyzing amino acid biosynthesis from OAA (*asd*, *dapA* and *dapD*) and decreased expression of genes catalyzing amino acid biosynthesis from α-ketoglutarate (*gdh*, *argD* and *argG*) can explain this phenotype (40).

### 2.3 Flux analysis

The distribution of metabolic flux through various metabolic networks plays a key role in determining biocatalyst behavior. Understanding the metabolic pathways required for production of the target compound and controlling the flux through these pathways can be enormously helpful in strain design and modification (49, 63). Fluxomics is widely used in metabolic engineering (10, 30, 70), as it not only provides a general view of the distribution of carbon throughout the metabolic network, but also quantifies intracellular metabolite turnover rates for specific metabolic pathways. Hence the information from comparing metabolic flux between control regulation and functional regulation can be assessed as guidelines for manipulating metabolic phenotype (37).

Flux balance analysis (FBA) predicted an optimal metabolic pathway in *E. coli* for succinic acid production. It was found that the pyruvate carboxylation pathway should be used rather than phosphoenolpyruvate carboxylation pathway (42). Based on the genome-scale *E. coli* stoichiometric model iJR904 and applied *in silico* optimization, the estimated maximal succinate yield was 1.6 mol succinate/mol glucose. Then a combination of *in silico* optimization and metabolic flux analysis identified three potential target genes for improving succinic acid production, including the glucose phosphotransferase transport system (PTS), pyruvate carboxylase, and the glyoxylate shunt. Genetic modification of these targets enabled higher succinate yields: 1.29 mol succinate/mol glucose, relative to the 0.15 mol/mol observed with the parent strain (76). Moreover, a powerful combination of genetic inventory and flux balance analysis has been demonstrated. Specifically, it was desirable to compare the central carbon metabolism of the succinate producer *Mannheimia succiniciproducens* to *E. coli* in order to find candidate genes for increased succinate production. Metabolic pathways that exist in *E. coli* but not in *M. succiniciproducens* were considered to drive metabolic flux away from succinic acid formation. Five genes, including *ptsG* (component of the phosphotransferase system), *pykAF* (pyruvate kinases), *mqo* (malat:quinone oxidoreductase), *sdhABCD* (succinate dehydrogenase), and *aceBA* (glyoxylate shunt enzymes), were found in *E. coli* but not in *M. succiniciproducens* and were selected as potential target genes for deletion. A flux balance analysis based on a genome-scale metabolic model of *E. coli* was used to select the optimal gene deletion combinations, and predicted deletion of *ptsG* and *pykAF* was promising, where the *ptsG* and *pykAF* deletion strains had a 100-fold higher succinate production rate than the wild type strain (41).

^13^C-based metabolic flux analysis is also a useful way to investigate metabolism *in vivo* (63, 78). The purpose of ^13^C-labeling is to investigate the operation of central metabolic pathways using labeled precursors. The distribution of these labeled carbons within downstream metabolites is determined by gas chromatography-mass spectrometry (GC-MS) or nuclear magnetic resonance spectroscopy (NMR), and additional constraints on the metabolic network are used to calculate the intracellular flux distribution (15, 19, 37, 70). In *S. cerevisiae*, L-malic acid is synthesized from pyruvate followed by reduction of OAA to malate (60). Genetic modifications which aimed to drive flux through this pathway were conducted in *S. cerevisiae*, including overexpression of native pyruvate carboxylase, cytosolic malate dehydrogenase and malate transporter from *Schizosaccharomyces pombe*. After genetic modification, the highest malate production was obtained with titers up to 59 g liter^-1^. Then, a^13^C-NMR-based metabolic flux analysis performed on the modified strains demonstrated that the flux distribution was consistent with involvement of pyruvic oxaloacetic acid pathway (82).

## 3. Engineering tolerance to product toxicity

Product toxicity is a pervasive problem in the metabolic engineering of microbial biocatalysts for economically viable production of biorenewable fuels and chemicals (1, 33, 53, 64). Specifically, the growth and metabolism of the biocatalyst can be inhibited at high product concentrations, limiting the amount of product formed. Historically, this problem is addressed through the use of metabolic evolution, as described below. However, an understanding of the mechanism of toxicity can enable rational engineering efforts to mitigate this problem (11, 33, 77). Omics analysis, as described above and reverse engineering of evolved strains can aid in understanding the toxicity mechanism.

Carboxylic acids have been reported to be toxic to microbes, possibly due to membrane disruption and perturbed metabolic pathway by cytosol acidification (1, 4, 12, 43, 45). Directed metabolic evolution serves to select for beneficial mutations by continuously culturing the cells under selective pressure (13). Acetic acid released from hydrolysis of lignocellulose is a strong inhibitor to microbes during production of chemicals from plant biomass (58). Two evolutionary strategies have successfully selected strains with acetic acid tolerance in *S. cerevisiae* (79). The first strategy was to culture the yeast cells in increasing concentrations of acetic acid while maintaining the pH at 4. The second strategy was conducted by prolonged anaerobic continuous cultivation without pH control. In this strategy, selective pressure for acetic acid tolerance was generated by acidification from ammonium assimilation. The evolved strains from both methods showed improved tolerance to acetic acid after 400 generations.

Transcriptome analysis is another useful tool to identify target genes for further strain development by comparing the expression profiles between strains with the acid-adapted and unadapted phenotype. The mechanism for carboxylic acid-tolerance has been extensively investigated in *S. cerevisiae* from genome-wide response by transcriptome analysis. Using global phenotypic analysis and transcriptional profiling, many genes related to weak acid resistance in *S. cerevisiae* have been identified to be regulated by Msn2p/Msn4p (66). A transcriptome analysis to investigate carboxylic acid toxicity (sorbate, acetate, propionate and benzoate) in *S. cerevisiae* identified 14 genes as up-regulated in response to all acids. Genes related to cell wall, such as *SPI1* encoding a glycosylphosphatidylinositol-anchored cell wall protein and *YGP1* encoding cell wall-related secretory glycoprotein, and membrane transport process were reported as overrepresented in this dataset (1), and Pdr12p is also up-regulated in response to sorbate, propionate and benzoate. Pdr12p transports weak acid anions from the cytosol by energy-dependent export (28). Furthermore, transcriptome responses to octanoic acid and decanoic acid in *S. cerevisiae* revealed that the expression of transporters such as Pdr12p and Tpo1p is important for detoxification of octanoic acid by exporting it out of cells. Decanoic acid resistance involved Tpo1p, genes related to the beta-oxidation pathway and ethyl ester synthesis. Note that both carboxylic acids activated oxidative stress genes (43). In addition, transcriptome anaylysis of the acetic acid response in *S. cerevisiae* showed that 80% of the acetic acid-activated genes were directly or indirectly regulated by Haa1p. Among these genes, the deletion of *HRK1*, which encodes a protein kinase dedicated to the regulation of membrane transporter activity, resulted in the increased acetate accumulation in acid-stressed cells and increased susceptibility to acetic acid (52).

Bacteria can detect environmental stress by sensor proteins, which are regulated by various transcription factors. A mathematical method, Network Component Analysis (NCA) based on known connectivity between transcription factors (TF) and genes, was applied in *E. coli* to analyze the dynamics of the activities of various TFs based on transcriptome profiles. Kao *et al* used NCA of 16 TFs to estimate transcription factor activities (TFA) during the transition from glucose to acetate (36). They found that the activities of TFs regulating genes for amino acid biosynthesis, nucleotide biosynthesis and carbon source transition were disturbed.

Metabolic flux analysis is also a useful tool in identification of the mechanism of inhibition. For example, a recent metabolic flux analysis of *E. coli* during octanoic acid challenge (Fu et al., in preparation) revealed decreased flux through pyruvate dehydrogenase and the TCA cycle, possibly due to the redox imbalance caused by membrane damage.

Recently, a combination of directed evolution, transcriptome analysis and reverse engineering constructed a succinate-tolerant *E. coli* strain (39). Wild-type *E. coli* W3110 was continuously cultured in a gradually increasing concentration of succinate for 9 months, at which the succinate concentration was 0.592M. The final evolved strain DST160 showed higher tolerance than the wild-type strain under the same succinate stress: in the presence of 0.592M succinate, DST160 showed a growth rate of 0.20 h^-1^, a 10-fold improvement relative to the wild-type strain value of 0.02 h^-1^. Comparative profiling by DNA microarray and quantitative PCR between DST160 and wild type W3110 showed that genes related to active transport and biosynthesis of osmoprotectants were upregulated. Furthermore, expression of *ygjE*, a putative succinate antiporter, and *betA*, for betaine biosynthesis, in non-adapted *E. coli* increased growth rate under succinate stress.

## 4. Combination of directed evolution and genetic engineering

While genetic manipulation and metabolic evolution are both while useful on their own, these tools become especially powerful when used together. Here, we review the examples where these two strategies have been successful combined to produce malate, succinate and lactate.

One such example is the alternating use of targeted gene deletion and growth-based metabolic evolution conducted in *E. coli* to improve the production of succinate and malate in mineral salts media (31). The first component of this strategy was to eliminate formation of lactate, ethanol and acetate by deleting *ldhA*, *adhE* and *ackA*, respectively. This left the malate and succinate pathway as the primary route for NAD^+^ regeneration and ATP production under fermentative conditions. Then the resulting strain KJ012 was evolved in growth-based selection in order to simultaneously select for improved growth and therefore, improved carboxylic acid production. The evolved strain was further improved by deleting genes involved in byproduct formation (*focA*, *pflB*, *poxB* and *mgsA*) and growth-based evolution was again used to generate two strains (KJ060 and KJ073) with production of 622-733mM succinate, and one robust malate strain producing 516 mM malate. Moreover, further study in two robust succinate producing strains KJ060 and KJ073 found two mutations responsible for their phenotypes. One is a promoter mutation in *pck*, leading to increased expression of PEPCK, increased ATP formation and therefore increased succinate production. The second mutation was a frameshift mutation within *pstI*, inactivating the PTS system. In this case, PTS-mediated glucose uptake was replaced by increased expression of galactose permease (*galP)* and glucokinase (*glk*) (84, 86).

The same engineering scheme was also successfully applied for lactate production (88). Deletion of the pathways for ethanol (*adhE*), acetate (*ackA*) and the *Z. mobilis* homoethanol pathway from *E. coli* KO11 generated strain SZ110. This left lactate production as the only method for NAD^+^ regeneration during fermentative growth. Then a growth based -evolution was performed on SZ110, resulting in strain SZ132. Further deletion of other foreign genes resulted in lactate-producing strain SZ186. Both SZ132 and SZ186 can produce 667-700 mM lactate in mineral salts medium. Further improvement from SZ186 by eliminating co-product formation and further metabolic evolution in mineral salts medium with glucose generated strain SZ194, with the production of 1.2M lactate from 12% glucose with addition of 1mM betaine as osmoprotectant (87).

## 5. Summary and Outlook

Developing fermentative processes that can provide biorenewable sources of bulk chemicals in a manner that is economically competitive with petroleum-based processes is becoming increasingly attractive, important and feasible. Here we have highlighted existing projects that clearly demonstrate that metabolic engineering is a useful tool in developing these processes. Specifically, we have focused on existing projects for the production of malate, lactate and succinate. Previous successes have also been reported for acetate, pyruvate, hydroxyacids and butanol (3, 16, 17, 29) and many groups are currently working on production of longer-chain and medium-chain carboxylic acids (C5 and C6) (44, 80, 83).

Metabolic engineering in the form of overexpression of key pathway genes, as well as deletion of competing pathways, has proved quite effective for improving carboxylic acid production. Omics analysis has also been indispensable in the selection of non-obvious metabolic engineering targets. Improved tolerance to carboxylic acids is a key aspect of this area that needs further attention to enable production of these chemicals at higher titer. It is also clear that the cell membrane will be a promising target for future metabolic engineering. Furthermore, efflux pumps which can export the carboxylic acids outside the cells will be useful for improve the tolerance. In the future, a combination of synthetic technology with current metabolic engineering information is expected to engineer a robust biocatalyst to produce biorenewable chemicals in place of petroleum.
